# Mitochondrial Quality Control: A New Perspective in Skeletal Muscle Dysfunction of Chronic Obstructive Pulmonary Disease

**DOI:** 10.14336/AD.2024.1129

**Published:** 2025-12-05

**Authors:** Yanxia Song, Xiaoyu Han, Yingqi Wang, Kangxia Li, Huanping Li, Yizhu Tian, Xiaoqing Ma, Weibing Wu, Jihong Wang

**Affiliations:** ^1^School of Exercise and Health, Shanghai University of Sport, Shanghai, China.; ^2^School of Rehabilitation Science, Shanghai University of Traditional Chinese Medicine, Shanghai, China.; ^3^School of Athletic Performance, Shanghai University of Sport, Shanghai, China.

**Keywords:** COPD, mitochondrial quality control, mitophagy, muscle atrophy, myogenesis

## Abstract

Skeletal muscle dysfunction (SMD), one of the extrapulmonary complications in patients with chronic obstructive pulmonary disease (COPD), considerably influences patient prognosis. Mitochondria regulates their dynamic networks through a mitochondria quality control (MQC) mechanism, involving mitochondrial biogenesis, mitochondrial dynamics, and mitophagy. The MQC is crucial for mitochondrial homeostasis and health, and disruption of it can lead to mitochondrial damage, which is a key factor in the structural and functional impairment of skeletal muscle in COPD. The mitochondria in the skeletal muscles of these patients undergo changes, mainly including decrease in mitochondrial density and biogenesis levels, imbalanced mitochondrial fission and fusion, and altered mitophagy status. However, the potential mechanisms linking MQC to the damaged structure and function of skeletal muscles in COPD have not been fully clarified. Therefore, this review highlights the effects and potential pathways of the MQC system on the dysfunction of skeletal muscle (muscle atrophy, impaired myogenesis and regeneration, and aerobic endurance) in patients with COPD, and summarizes potential interventions targeted MQC, intending to provide a theoretical basis for further research on COPD, improve SMD, and enhance the quality of life.

## Introduction

Chronic obstructive pulmonary disease (COPD) being a growing health concern, is one of the three major causes of death globally, especially in developing countries [1], posing huge economic and social burden [2]. Owing to the aging of global population and the rising exposure of associated risk factors, the prevalence and burden of COPD will continue to grow in the future years [3]. COPD is defined by GOLD 2024 as a “heterogeneous lung condition characterized by chronic respiratory symptoms (dyspnea, cough, expectoration, exacerbations) due to abnormalities of the airways (bronchitis, bronchiolitis) and/or alveoli (emphysema) that cause persistent, often progressive, airflow obstruction” [4]. Beyond respiratory issues, patients with COPD usually suffer from at least two extrapulmonary complications [5, 6], including osteoporosis, skeletal muscle dysfunction (SMD), and cognitive deficit et al., which lead to the deterioration of the patients.

SMD is often linked to higher morbidity and mortality in patients with COPD. Specifically, there is a decrease in muscle mass and endurance, in turn contributing to poor athletic performance, impairing the capacity for executing daily activities and their quality of life [7]. Muscle mass is indirectly related to the patient outcome and prognosis. The maximal voluntary contraction force of the quadriceps (QMVC) and cross-sectional area (CSA) of the mid-thigh muscle on CT scan are effective predictors of mortality to moderate to severe COPD [8, 9]. Furthermore, decreased quadricep strength is connected to increased mortality in patients with COPD and offer prognostic information [10]. Patients with COPD have reduced muscular endurance and oxidative capacity. Animal experiments have shown that muscle repair and regeneration are impaired in COPD mice modeled by inflammation [11]. These effects contribute to decreased exercise capacity or exercise intolerance in patients. The current mechanisms of SMD in COPD may include dysregulation due to decreased physical activity levels, systemic inflammation, oxidative stress, mitochondrial damage, hypoxemia, and steroid use, but these remain unclarified [7, 12, 13].

Mitochondrial damage is a critical cause of SMD in COPD. Mitochondria are the main source of intracellular ATP in skeletal muscle and the primary target of reactive oxygen species (ROS), making them particularly susceptible to damage during physiological adaptations and stressful conditions [14]. Quality control systems recognize and address mitochondrial dysfunction when mitochondria are damaged [15]. Mitochondrial biogenesis, mitochondrial dynamics, and mitochondrial degradation pathways (mitophagy) constitute the mitochondrial quality control system (MQC), and these mechanisms act at the molecular or organelle level and work synergistically to maintain a healthy mitochondrial population. These MQC pathways do not exist in isolation [16, 17]. Mitochondria are dynamic organelles with significant plasticity, and changes in morphology are mediated by mitochondrial dynamics (fission and fusion). Fusion between healthy and impaired mitochondria maintains overall mitochondrial fitness by diluting damaged organelles into the healthy mitochondrial network, whereas mitochondrial fission separates dysfunctional mitochondria from the healthy mitochondrial network and triggers the next step in the mitochondrial phagocytosis degradation mechanism [18]. Through these mechanisms, skeletal muscle cells coordinate mitochondrial morphology and function and maintain mitochondria at homeostatic levels to maintain optimal muscle function.

MQC in skeletal muscle is disrupted in patients with COPD, functional mitochondria are destroyed, and mitochondrial damage occurs. Mitochondrial biogenesis in skeletal muscle is impaired of those with COPD [19-21]. Tan et al. [22] observed that smoke-induced mitochondrial division was enhanced in C2C12 adult muscle cells of COPD mice, the kinetic fission process was over-enhanced, and the perturbation of the dynamics led to a decrease in cellular activity. Mitophagy process has a key role in COPD muscle atrophy [23]. These all suggest that the MQC system is perturbed in skeletal muscle of COPD patients, causing mitochondrial damage. However, the exact role of mitochondrial damage in the pathogenesis of COPD-related SMD is still unclear. In this paper, we clarify the impact of mitochondrial damage on SMD in COPD and the potential mechanisms from the perspective of mitochondrial quality control mechanisms. This information can contribute to the determination of pharmacological targets for regulating mitochondrial function and preventing muscle loss and development of novel therapeutic modalities that can improve the outcome and quality of life of patients with SMD-associated COPD.

## Skeletal muscle dysfunction in COPD

SMD, a predictor of death in COPD [24], mainly shows a decreased muscle mass and endurance. These are connected to structural and biological alternations in the muscles of COPD patients, thus affecting their muscle function (strength and exercise capacity).

Structural changes in the muscles of COPD mainly involve wasting muscle mass and reduced capillary density surrounding skeletal muscle. Weight loss (decreased BMI) is a common symptom in patients with COPD, and even fat-free mass depletion probably occur in normal-weight patients [25]. Muscle atrophy also reflects a loss of muscle mass, with a prevalence of about 21.6% in patients with COPD [26]. Reduced thigh diameter versus microscopic quadriceps muscle fiber CSA was observed in patients with COPD [27], and the degree of muscle mass loss in the thighs accounted for a greater proportion of the total body relative to the whole body, suggesting that muscle weakness is preferentially distributed in the lower limbs [28]. In addition to muscle atrophy, impaired skeletal muscle regeneration may also lead to decreased muscle mass. Patients with COPD have relatively low levels of muscle repair after injury [29]. In addition, Satellite cells cultured from skeletal muscle of COPD patients showed senescent features, suggesting impaired muscle regeneration capacity [30]. Satellite cells isolated from COPD muscle biopsies showed delayed activation, considerably reduced MHC accumulation at the terminal stage of the myogenic differentiation, and inability of adult myoblasts to fuse, suggesting defects in myotube maturation. Additionally, the amount of capillaries around a single muscle fiber is reduced in COPD patients compared with healthy individuals, suggesting that COPD patients have reduced muscle capillary formation and are more susceptible to contraction fatigue [31]. Eliason et al. [32] investigated the muscle capillary interface (a sensitive indicator of changes in the capillary network of limb muscles) and revealed a positive relationship between the degree of airflow obstruction in type I and type IIa fibers and the CFPE index, and muscle capillarization decreases with increasing disease severity.

Several biological changes occur within COPD skeletal muscle, including shifts in muscle fiber type, mitochondrial dysfunction and reduced activity of key oxidative enzymes. In limb muscles, no significant differences were noted between patients with early mild stable COPD and controls in either fiber type proportions or CSA, but a decline in the proportion of oxidative type I fibers and a predominance of type II fibers were observed in patients with advanced COPD [33], showing a transformation in the phenotype of muscle fiber occurs. Type II fibers have fewer mitochondria are less oxidative, and are more susceptible to fatigue than type I fibers [34]. Besides, in COPD, mitochondrial structural and functional abnormalities are also important biological features, mitochondrial dysfunction in patients with COPD and smoke-induced COPD mice are promoted by reduced mitochondrial gene transcription in the skeletal muscles, low biogenesis drive, mitochondrial swelling, disruption of the morphological structure of the typical filamentous mitochondrial network, shortening of the morphology, cristae absence, most of which show fragmentation and vacuolization, abnormal MQC, impaired mitochondrial activity and respiratory chain complexes [22, 23, 35, 36]. Patients with COPD have reduced quantity of mitochondria and key oxidative enzyme activity, such as citrate synthase and succinate dehydrogenase, in the quadriceps. Moreover, the expression of genes for enzymes vital in glycolysis and oxidative phosphorylation is suppressed, resulting in a reduction in isometric endurance in the quadriceps, which is consistent with the typical reduction of type I fibers seen in the quadriceps [37].

Functionally, patients with COPD exhibit muscle weakness and decreased muscular endurance. In patients with early COPD, the maximum isometric contraction force of the quadriceps muscle of the lower limb is reduced by 20%-30%, and the quadriceps muscle strength is reduced by an average of 25% compared with that of healthy people [38]. Similar to lower extremities, upper extremity strength is affected in COPD, and patients have a reduced ability to generate force in upper extremity muscles [39]. Grip strength decreases significantly in patients with COPD in the advanced stages of this disease, and lung function and prognosis can be predicted by grip strength testing [40]. Alterations in the structure and biology of muscle fibers in COPD can contribute to reduced skeletal muscle endurance. Gosker [41] et al. detected a significant muscle fiber type shift, specifically, the proportion of type I fibers is declined and that of type II fibers is elevated in the lateral femoral muscle in patients with COPD. Besides, the amount of myofibrillar mitochondria and oxidative enzymes is reduced, which results in a decrease endurance and resistance to fatigue in the muscles of COPD. Decreased skeletal muscle strength also leads to exercise intolerance in patients with COPD. 6MWT result is decreased in COPD [42]. Adami [43] et al. found that the oxidative capacity in the muscles of both the upper and lower extremities was reduced in COPD. A meta-analysis that included 21 studies involving 728 COPD patients and 440 healthy subjects found that quadriceps endurance was diminished in patients with COPD [44].

## Mitochondrial Quality Control

**Figure 1.**
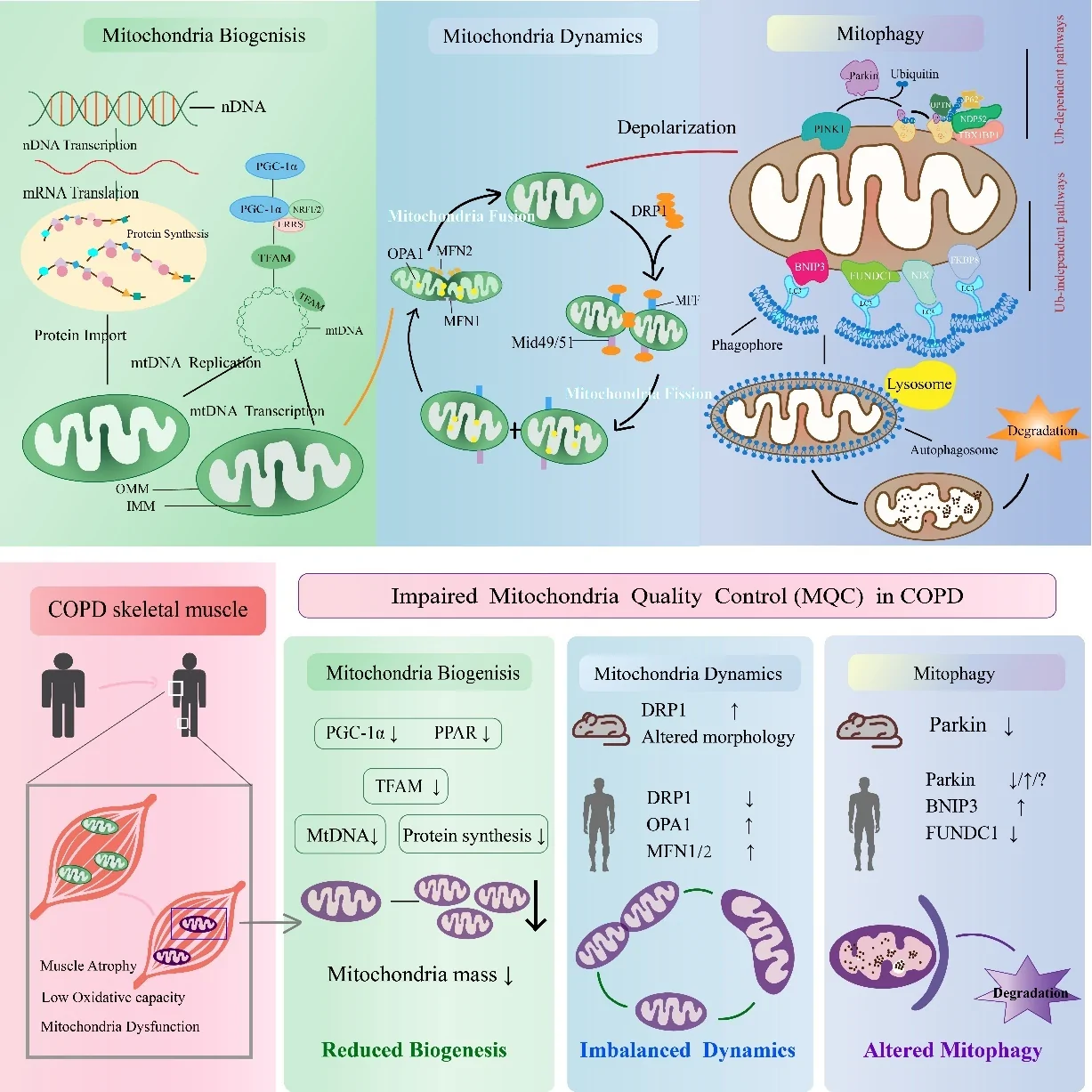
**MQC system and MQC changes in COPD**. MQC requires the coordination of mitochondrial biogenesis, dynamics and mitophagy. Mitochondrial biogenesis is coordinated between mtDNA and nDNA, and the factors responsible for regulating mitochondrial biogenesis are mainly the PGC-1family and the NTF family, which includes NRF-1 and NRF-2. Mitochondrial dynamics includes fission and fusion. The OMM fusion is mainly modulated by MFN1 and MFN2, and OPA1 is for IMM fusion. DRP1 is the key regulatory factor of fission, which requires junction proteins (MFF, MiD49 and MiD51) at the OMM. Mitophagy, the specific elimination of damaged mitochondria, is controlled by two pathways, one ubiquitin-dependent and the other receptor-mediated(ubiquitin-independent). The ubiquitin-dependent pathway mediated by the pathway consisting of PINK1 and Parkin is one of the most classical pathways, which can be initiated by mitochondrial depolarization. Apart from the ubiquitin-dependent pathway, specific proteins localized to the OMM, such as BNIP3, NIP3 (also known as NIX), FUNDC1, FKBP8, and BCL2L13, also act as receptors to mediate mitophagy. In skeletal muscle of patients with COPD, mitochondrial alterations are manifested by reduced biogenesis, imbalanced mitochondrial dynamics, and altered mitophagy. Thus, leading to impaired MQC.

The structural and functional integrity of mitochondria are essential for the maintenance of cellular life activities, play a key role in metabolism and signaling pathways, and are related to several diseases [45]. The MQC includes mitochondrial biogenesis, mitochondrial dynamics and mitophagy [46] (Fig. 1).

Mitochondria form a dynamic network in most cell types, constantly remodeling through organelle fusion and fission [47]. The coordination between the generation of new mitochondria (biogenesis) and the clearance of defective mitochondria (mitophagy) remodel and shape the mitochondrial network [48, 49]. In addition, mitochondrial dynamics is the link connecting biogenesis and mitophagy [46],and can regulate the subcellular localization of mitochondria [50].

## Mitochondrial Dynamics

Mitochondria fission and fusion are two key processes in the mitochondrial lifecycle and are called “mitochondrial dynamics”. The balance between these opposing events regulates their quantity, size, and position [51]. Mitochondrial function is closely related to its morphology, and morphology varies in physiological and pathophysiological conditions [50]. Mitochondrial morphology is formed by continuous fusion and fission of the outer and inner membranes (OMM and IMM) [52]. Mammalian OMM fusion is regulated by two membrane GTPases present on the OMM: mitochondrial protein Mitofusin 1 (MFN1) and mitochondrial protein 2 (Mitofusin 2, MFN2) [53]. Optic nerve atrophy protein 1 (OPA1), a dynamin-like GTPase located in the IMM, is required for IMM fusion [54]. The specific phospholipid components of the mitochondrial inner membrane can also mediate IMM fusion [55]. OPA1-dependent IMM fusion is dependent on MFN1 but not on MFN2, suggesting potential interaction between the two membranes in fusion [56]. Mitochondrial fission is a multistep process that is largely dependent on the cytoplasmic GTPase (Dynamin-related protein1 (DRP1)), which is recruited from the cytoplasm, forms a helix around the mitochondria, and shrinks to sever the IMM and OMM [57]. DRP1 lacks the PH domains for membrane phospholipids bonding, and junction proteins are needed for its recruitment at the OMM. The tail-anchoring proteins mitochondrial fission factor (MFF) and mitochondrial dynamin 49 and 51 (MiD49 and MiD51) are receptors for DRP1 in mammals [58, 59]. The mechanism of IMM division has not been extensively studied, and IMM division still occurs without DRP1 or OMM contraction [60], suggesting the existence of a fission mechanism independent on DRP1. Chakrabarti [61]et al. showed that IMM contraction may be calcium-dependent and occurs at the mitochondria-endoplasmic reticulum contact site.

## Mitochondrial biogenesis

In response to higher energy demands and other reactions, mitochondrial biogenesis is the mechanism of augmenting the mass and quantity of mitochondria. Mitochondrial biogenesis includes (1) “synthesis of the IMM and OMM” (2) “synthesis of mitochondrial-encoded proteins” (3) “synthesis and import of nuclear-encoded mitochondrial proteins” (4) “mtDNA replication and transcription” [62]. Mitochondria are semiautonomous organelles that synthesize small amounts of proteins by transcribing and replicating mtDNA-encoded genes, whereas a huge proportion of the mitochondrial protein is nuclear-encoded, synthesized on cytoplasmic ribosomes, and finally sorted and imported into the appropriate location within mitochondrial. Protein precursors in the cytoplasm require translocases to be imported into the mitochondria. There are 4 major membrane protein translocase complexes recently [63]:TOM (outer membrane translocase), TIM (inner membrane translocase), PAM (presequence translocase-associated motor), and SAM. Mitochondrial biogenesis requires coordination of mitochondrial genes (mtDNA) and nuclear coding genes (nDNA) [64],and it is dependent on synchronized interactions between mitochondrial and nuclear factors. Regulatory pathways responsible for regulating mitochondrial biogenesis include those defined by transcriptional coactivators (e.g., Peroxisome proliferator-activated receptor-γ (PPAR) coactivator 1 (PGC-1)) and nuclear transcription factors (including nuclear respiratory factors 1 (NRF-1) and 2 (NRF-2)). [65, 66]. The PGC-1 family has been a central regulator of mitochondrial biogenesis and mitochondrial metabolism [67],which consists of PGC-1α, PGC-1β and PRC [68]. PGC-1α is a key regulator of mitochondrial biogenesis and maturation and binds to a range of nuclear transcription factors, including NRF1/2, estrogen-related receptor α (ERR-α), and PPAR, increasing the expression of mitochondrial transcription factor A (TFAM) and many mitochondrial respiratory chain genes, and mediating mitochondrial biogenesis [69]. TFAM is a direct target of NRF-1 and NRF-2 and a terminal executor of mtDNA transcription and replication, contributing significantly to these genetic activities [70]. PGC-1β is also essential for the modulation of mitochondrial biogenesis and is additionally involved in mitophagy and apoptosis [71]. PPARγ-associated coactivator 1 (PRC) may also involve in the regulatory control of mitochondrial biogenesis, and silencing PRCs reduces the expression of nearly 50 mitochondria-related genes, such as respiratory chain subunits, mitochondrial protein inputs, and assembly factors [72].

## Mitophagy

Mitophagy is a process for selectively removing impaired mitochondria, targeting and delivering them to lysosomes for degradation [73], facilitating the expansion and formation of a functional mitochondrial network [74],and effectively removing damaged mitochondria to aviod apoptosis [75]. There are two types of mitophagy pathways, one ubiquitin-dependent and the other receptor-mediated. The ubiquitin-dependent pathway consisting of PTEN-induced putative protein kinase 1 (PINK1) and the Parkin (E3 ubiquitin ligase) is one of the most classical pathways in mammals [76]. The decline or loss of mitochondrial membrane potential serve as a initiating signal for mitophagy, resulting in the failure for PINK1 to enter or normally transported the OMM, accumulating on damaged mitochondria thereby recruiting Parkin in the cytoplasm [77]. Parkin, once recruited, promotes their dissociation from the mitochondrial network and in promoting ubiquitination of mitochondrial proteins ultimately leads to autophagic degradation of these organelles [78, 79]. Parkin primarily mediates the formation of two types of polyubiquitin chains, a lysine K48 bond associated with proteasomal degradation of the substrate and a lysine K63 bond associated with autophagic degradation. The remaining six “atypical” ubiquitin chains can also be formed through Parkin, and each of the atypical ubiquitin chains has a different function [80, 81]. Once mitochondria are ubiquitinated, they are identified by adaptor proteins through their ubiquitin-binding domains. There are five types of adaptor proteins [82]:next to BRCA1 Gene 1 Protein (NBR1), Nucleolar Dot Protein 52 (NDP52), Optic Nerve Phosphatase (OPTN), Sequesterosome-1 (SQSTM1/p62), and tax1 Binding Protein 1 (TAX1BP1). When autophagy receptors are recruited to mitochondria, NDP52 and Optineurin [83] recruit ULK1, DFCP1, and WIPI1 to the proximal mitochondria, the autophagy receptor then binds to ubiquitylated substrates and LC3-encapsulated phagocytes to mediate autophagy. Besides the ubiquitin-dependent pathway, several proteins localized at the OMM, such as BNIP3, NIP3 [84] (also known as NIX), FUNDC1 [85], FKBP8 [86] and BCL2L13also act as receptors to mediate mitophagy [73, 87], which can target dysfunctional mitochondria, and then recruit autophagosomes for degradation through engagement with LC3 and GABARAP [88, 89].

## Mitochondrial Quality Control in COPD Skeletal Muscle Dysfunction

Mitochondrial dysfunction is strongly connected to the alteration in muscle structure and metabolic dysfunction [90]. Skeletal muscle is the main place of metabolic activity for carbohydrates, lipids and protein. The Dynamic regulation of mitochondria in skeletal muscle is fundamental to ensure the metabolic requirements [91]. Disturbance in the MQC of skeletal muscle in COPD may lead to muscle atrophy, impaired muscle regeneration and oxidative capacity.

## Mitochondrial Quality Control and COPD Skeletal Muscle Atrophy

Robust evidence in skeletal muscle supports that dysregulated mitochondrial dynamics lead to muscle wasting. Changes in mitochondrial dynamics in the muscle of COPD are controversial. Gosker [41]et al. found no variation in mitochondrial size in the quadriceps, indicating that mitochondrial dynamics may not be altered. Decreased DNM1L protein and gene expression and increased fusion proteins OPA1 and MFN1 proteins [19] were found in quadriceps muscle biopsies of patients with COPD, which demonstrate that mitochondrial fission may be slightly reduced (Fig. 1). Smoke-induced COPD mice had an increased number but decreased activity of mitochondria, reduced mitochondrial cristae, vacuolization of mitochondria, and shortened or fragmented morphology in the quadriceps muscle. These changes that are consistent with an increased expression of the DRP1 protein and the DRP1 mRNA, suggesting the enhanced fission in patients with COPD and can contribute to SMD [22, 92] (Fig. 1). None of these experiments quantified actual mitochondrial fission, and it is challenging to monitor and reflect the real state in mitochondrial dynamics at the level of gene expression alone. Quantitative imaging-based assessment of mitochondrial morphology and dynamics, utilizing the complete 3D mitochondrial network and extending it to 4D analysis, may provide valuable insights into changes in skeletal muscle dynamics in COPD [93]. Fission, fusion, and muscle mass control are closely related, and impaired mitochondrial fusion processes can lead to muscle atrophy when skeletal muscle MFN2 [94] and OPA1 [95] are absent. Song [96] et al. proposed that an imbalance in mitochondrial network dynamics is more deleterious than the absence or alteration of fusion fission events alone. Changes in mitochondrial dynamics in peripheral skeletal muscles in COPD are rarely studied, but what can be hypothesize is that there is an imbalance in mitochondrial dynamics and probably have a contributing effect on muscle atrophy and loss of skeletal muscle mass.

The harmonization between mitochondrial biogenesis and mitophagy maintains the steady turnover of the network. PPARα mRNA levels were remarkably decreased in skeletal muscle of patients with COPD and positively related to mitochondrial density, volume, and skeletal muscle Z-line width [21]. Besides, Remels [97] et al. proposed that disruption of the PPARα-PGC-1α-TFAM axis in skeletal muscle of patients with malignant COPD lowers PPAR-α mRNA expression and a low drive for mitochondrial biogenesis is associated with reduced skeletal muscle mass in patients with COPD (Fig. 1). Activating PGC-1α to induce mitochondrial biogenesis has been demonstrated to inhibit muscle atrophy in different diseases, and PGC-1α overexpression provides protection against proteolytic metabolism and muscle atrophy [98]. In addition to lower biogenesis, mitophagy is altered in patients with COPD, and this change may cause SMD (Fig. 1). Gouzi [99] et al. found that myotube mitochondrial autophagic flux, autophagy marker expression, and autophagy signaling was enhanced in cultured myotubes from quadriceps femoris muscles of patients with COPD as compared with the healthy population. Moreover, Leermakers [19] et al. exhibited the expression of Parkin gene and protein were higher in skeletal muscle of patients with COPD and negatively correlated with FEV1% prediction. Receptor-mediated mitophagy-associated protein BNIP3L protein levels were elevated, but FUNDC1 protein levels were lower (this may be related to the overall low content of mitochondria). Additionally, studies found that mitophagy is deficient in skeletal muscle of patients with COPD. Akihiko Ito [23] et al. proposed that the Parkin gene transcription was considerably reduced in the gluteus maximus of patients with COPD, and Parkin protein and its mRNA levels were also reduced in myotubular cells cultured in vitro in CS-induced COPD mice, and the mitochondria of these myotubular mitochondria were abnormally swollen and increased in fragmentation. There are four possible explanations for this: 1) Akihiko Ito et al. chose subjects who were approximately 85 years old, and their Parkin levels and mitochondrial autophagic fluxes may have been affected by aging [23]. 2) Different skeletal muscle fibers respond differently to mitochondrial perturbations, and Akihiko Ito et al. chose the gluteus maximus muscle rather than the quadriceps muscle, which may have affected the results. 3) Only the quantity of autophagosomes and the expression of autophagy-related genes were measured, but not the actual muscle autophagic flux in vivo. An elevated number of autophagosomes does not always correspond to enhanced autophagy, which could also result from impaired fusion of autophagosomes with lysosomes or lysosomal dysfunction. Therefore, a standardized method to monitor this cellular pathway is needed, such as “*Guidelines for the use and interpretation of assays for monitoring autophagy (4^th^ edition)”* [100]. 4) Different experimental subjects (animal models or human) may be at different stages of the disease, we believe that mitophagy may first be up-regulated in response to stress, creating the illusion of hyper-autophagy. Owing to the accumulation of autophagosomes, insufficient hydrolysis and down-regulation of biogenesis, mitochondrial depletion occurs if the body has been up-regulating mitophagy, and therefore the choice is made to down-regulate mitophagy.

## Mitochondrial Quality Control and COPD Impaired Myogenesis and Muscle Regeneration

Myogenesis is a key process controlling skeletal muscle development and homeostasis, and dysfunctional myogenesis leads to muscle dysfunction in COPD [101, 102]. MQC plays a crucial role in regulating myogenesis, and impairment of mitochondrial function hinders myogenic differentiation and muscle regeneration after injury. The mitochondrial biogenesis-involved genes such as PGC-1β, PRC, NRF-1, NRF-2, TFAM [103]were synchronously expressed with myogenesis-regulated genes such as MyoD and Myogenin. Skeletal muscles in which chloramphenicol blocked mitochondrial protein synthesis were poorly repaired, and small muscle fibers and increased amounts of connective tissue were detected [103]. These indicate that mitochondrial biogenesis is activated early in the muscle regeneration process and is involved in myogenesis.

Sin [74] et al. presented that mitophagy is an essential process for myogenic differentiation of adult myoblasts and impaired autophagy negatively affects myogenesis; they also showed that Dnm1L (encoding the DRP1 protein) mediates fission in mitochondrial dynamics during early myogenic differentiation is a key prerequisite for mitophagy, which is up-regulated during the early stages of differentiation but clears shortly thereafter. Tetrandrine [104] induced incomplete autophagic flux, which significantly impaired mitochondrial network remodeling and inhibited differentiation and regeneration of C2C12 myofibroblasts. Chronic inflammation-induced COPD mice exhibit signs of muscle atrophy and impaired muscle regeneration. Increased expression levels of Beclin1, a central regulator of autophagy, were found in satellite cells derived from these COPD mice [105], and autophagosome turnover was slowed down. The autophagy-inducing drug suberin promotes autophagosome turnover, replication capacity, and myogenic potential of muscle satellite cells (MuSCs) in COPD mice, and myogenesis improves after transplantation, suggesting that dysregulation of autophagy partially contributes to the dysfunction of myogenic regeneration in COPD. This beneficial impact of autophagy can be mediated through mitophagy and prevention of oxidative stress [106, 107].

Kim [108] et al. presented that DRP1 protein expression is upregulated during early differentiation of C2C12 myotubes and that DRP1 is rapidly translocated to the mitochondrial region. Inhibition of DRP1 by treatment with mdivi-1, a targeted inhibitor of DRP1 GTPase, reduced gene expression in myoblasts, inhibited myotube formation, and impaired myogenic differentiation. This indicates that DRP1- mediated mitochondrial fragmentation is a necessary step for successful myogenic differentiation. However, De Palma [109] et al. using specific nitric oxide (NO) synthase inhibitors, blocking of the NO/cGMP pathway enhances DRP1 activity, resulting in an excessive mitochondrial fragmentation and consequently delays myogenic differentiation, especially at early stages of differentiation. It is suggested that the overactivation or suppression of endogenous DRP1 leads to delayed myogenic differentiation and perturbations in mitochondrial dynamics prevent normal mitochondrial adaptation during muscle development. However, research on the connection between mitochondrial dynamics and myogenic differentiation and muscle regeneration in COPD skeletal muscles and MuSCs is limited. What is certain is that myogenesis and regeneration also require the regulation of mitochondrial dynamics.

## Mitochondrial Quality Control and Decreased Oxidative Capacity in COPD Skeletal Muscle

Skeletal muscle oxidative capacity is highly rely on the number as well as the activity of functional mitochondria, the decrease of oxidative capacity in lower extremity skeletal muscle is characteristic in COPD, reflecting pathological alterations in mitochondrial function [41, 110]. In the quadriceps of patients with COPD, mitochondrial density is diminished (both in number and area) [41], with lower enzyme activity [111] and decreased mitochondrial DNA replication [112], which is consistent with impaired mitochondrial biogenesis. Compared with healthy muscle, peripheral muscle of COPD patients showed lower levels of PGC-1α, lower mtDNA/nDNA ratios [112, 113], and reduced levels of TFAM proteins [20, 97]. Additionally, Konokhova [20] et al. found that the transcript levels of mitochondrial biogenesis signals were upregulated, including PGC-1α and TFAM, as well as PPAR, but the protein expression was reduced; notably, they observed decoupling between TFAM transcript levels and TFAM protein, which indicated that TFAM translation in COPD muscle is problematic, the mitochondrial biogenesis is insufficient. An 8-week pulmonary rehabilitation program of PUFA supplements considerably improved the peak exercise capacity and submaximal endurance time of patients with moderate-to-severe COPD, suggesting that fatty acid-induced activation of mitochondrial biogenesis by the PPAR pathway further enhances the training-induced mitochondrial response [114]. These show the low drive of mitochondrial biogenesis may be an explanation for the diminished oxidative capacity of skeletal muscle in COPD.

MQC is critical for muscle oxidative capacity, and over the past few decades, research mainly concentrate on impaired mitochondrial biogenesis in skeletal muscle from patients with chronic diseases [20, 97]. However, mitochondrial degradation process, that is, mitophagy, also influences the oxidative capacity. Autophagy [115] is necessary for exercise to induce phenotypic adaptation and oxidative capacity enhancement in skeletal muscle, potentially revealing a connection between mitophagy and skeletal muscle oxidative capacity. Leermakers [116] et al. mentioned that factors inducing the initiation of mitophagy in COPD may contribute to a decrease in muscle oxidative capacity, and the pathologic process of mitophagy is associated with impaired oxidative capacity. However, the specific mechanism underlying mitophagy and decreased skeletal muscle oxidative capacity in COPD is not clear because studies on mitophagy did not simultaneously test the oxidative capacity of skeletal muscle.

Furthermore, there is a significant diversity in mitochondrial morphology across different tissues, and mitochondrial shape in myofibers is fiber-type-dependent; in oxidative fibers, mitochondrial form a lattice-like network conformation with elongated structures in a parallel and perpendicular orientation; and the mitochondrial network in glycolytic fibers is branched and vertical to the muscle contractile axis and the I-band [15]. Mishra [117] et al. identified discrete mitochondrial structural domains within myofibers, the size of which correlates with the oxidative capacity of myofibers. The shape of these domains is controlled by mitochondrial fusion and fission. An RNA sequencing of muscle from participants in the *Study of Muscle, Mobility, and Aging* found that dynamics-related genes (DNM1L, OPA1) are positively correlated with peak VO2, maximal OXPHOS, and 400-meter walking speed, and revealed a potential relationship between kinetics and skeletal muscle oxidation levels [118]. These suggest that mitochondrial dynamics may regulate the distribution of mitochondria in skeletal muscle to correspond to the body's need for energy supply. An imbalance in mitochondrial dynamics may lead to a decrease in oxidative capacity.

We have to suggest that the current animal models and the different modeling methods are able to simulate some of the characteristics of COPD, but they have their limitations [119]. The different stages of disease progression replicated by different models, the anatomical structures differences from humans, the difficulty of modeling the more severe stages of COPD in some of the models, and the limited research reagents, etc. [120], considering which the study cannot exclusively rely on the results of a single animal experiment to make judgments.

## Possible mechanisms of mitochondrial quality control affecting skeletal muscle dysfunction in COPD

### Mitochondrial Quality Control and Muscle Protein Synthesis and Catabolism

**Figure 2.**
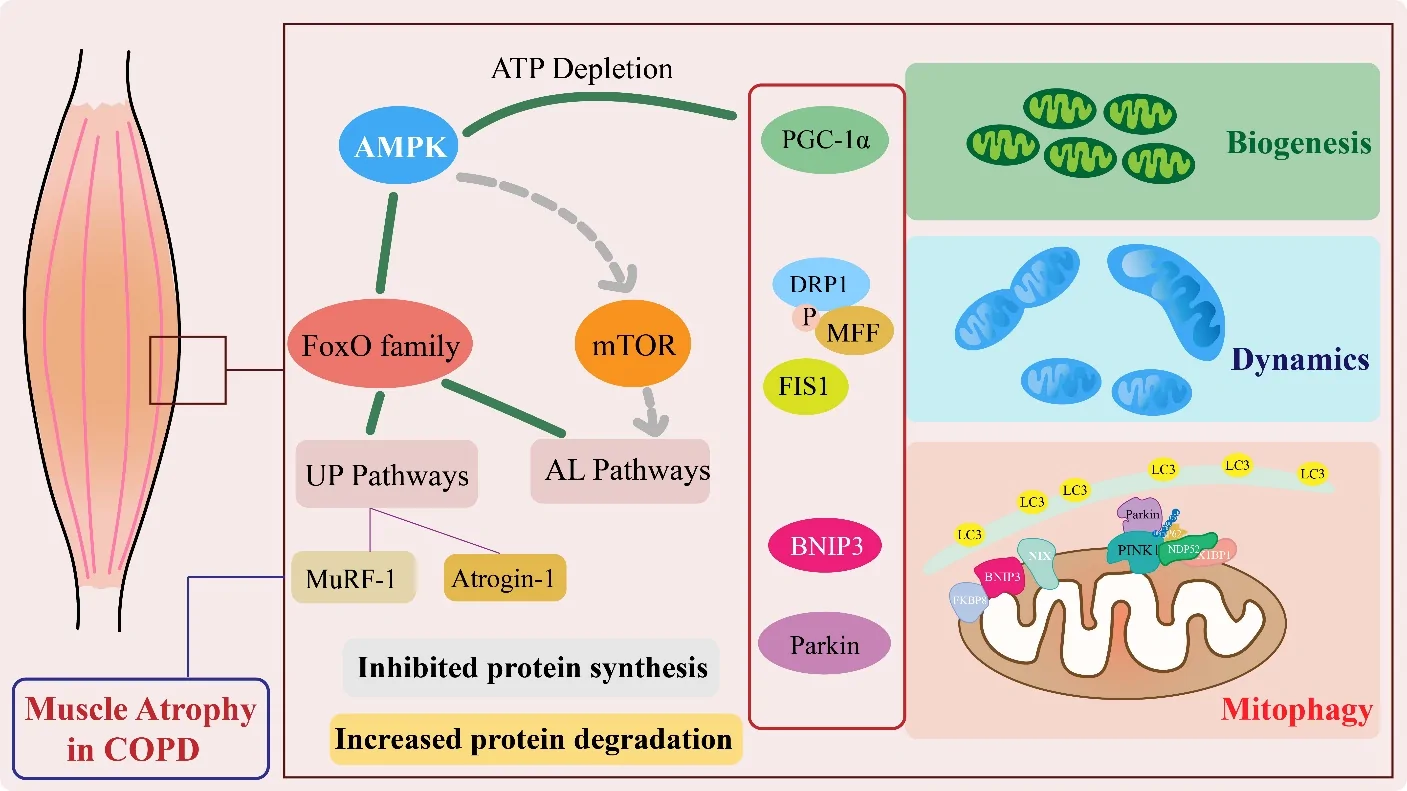
**MQC and muscle atrophy in COPD**. In pathological settings, the imbalance in mitochondrial quality control leads to altered levels of several genes and proteins such as PGC-1α, DRP1, Parkin, and BNIP3, which can activate AMPK. AMPK can inhibit protein synthesis and promote proteolysis in skeletal muscle. These result in muscle atrophy in COPD. In the figure, green lines are promotional, gray lines are inhibitory.

Excessive protein catabolism relative to synthesis is one of the signs of muscle atrophy, contributing to a loss of skeletal muscle mass, which is influenced by the relative intensity between protein synthesis and degradation. Intracellular protein degradation is mainly regulated by two pathways: the ubiquitin-proteasome (UP) pathway and the autophagy-lysosome (AL) pathway. [121, 122]. In UP pathway, two major E3 ligases, namely, muscle atrophy F-box (atrogin-1) and muscle RING Finger 1 (MuRF-1) are thought to be essential for muscle atrophy. In patients with COPD, the protein degradation is hyperactivated, and levels of atrogin-1 and MuRF-1 are elevated [121], which may lead to muscle atrophy (Fig. 2). The AL pathway is autophagy lysosome-dependent degradation of cytoplasmic components and is a vital part for maintaining protein turnover. A study identified the number of autophagosomes was elevated in the lateral femoral and tibialis anterior muscles (TIA) of COPD, and the degree of lipidation of the LCB3 protein was related to a reduced CSA of the thigh [123].

PGC-1α can regulate protein turnover by modulating the UP and AL pathways through the Forkhead box O-like family of transcription factors (FoxO). The knockdown of PGC-1α leads to muscle fiber damage in mice. PGC-1α inhibits FoxO3 binding to the atrogin-1 promoter, thereby reducing FoxO3-dependent atrogin-1 transcription [98]. High levels of PGC-1α in muscle significantly attenuated the up-regulation of MuRF-1 and atrogin-1 genes (the UP system), inhibited the induction of p62 (the AL system) in spent halibut muscle, and decreased the LC3II/LC3I ratio in mouse tibialis anterior muscle, and muscle CSA is higher in overexpressing mice than in wild mice [124, 125]. The activation of the AMPK/PGC-1α pathway is pivotal to regulate mitochondrial biogenesis in skeletal muscle, and AMPK can inhibit protein synthesis and promote proteolysis in skeletal muscle through inhibition of the mTOR pathway and activation of the FoxO-dependent pathway [126, 127](Fig. 2).

Parkin, a major mitophagy regulator gene, is crucial for skeletal muscle mass and may explain SMD in COPD. Gene transcription results in the quadriceps of patients with COPD showed considerably reduced levels of Parkin expression [128], and the knockdown of Parkin inhibits mitophagy and impairs mitochondrial turnover in mouse experiments and in vitro myoblast cell cultures, which further contributes to muscle atrophy and loss of muscle mass [129]. Mice overexpressing Parkin [130] have increased muscle fiber area and increased muscle mass. Parkin can regulate AMPK activity, which induces mitochondrial fragmentation by phosphorylating MFF, a receptor for DRP1-mediated fission, thereby initiating mitophagy [131] (Fig. 2).

Romanello [132] et al. found that excessive mitochondrial fission was adequate to lead to muscle atrophy in animal models, inhibited mitochondrial fission prevented muscle loss during denervation, and mitochondrial dynamics often acts in conjunction with mitophagy. Suppression of Fis1 or combined blockade with BNIP3 substantially protected against muscle loss in mice, and Fis1 and BNIP3 knockdown attenuated activation of the atrogin-1 and MuRF-1 promoters during fasting (Fig. 2). In addition, the inhibition of DRP1 decreases FoxO3-mediated autophagosome formation. This may be due to the activation of the AMPK-FoxO3 axis by mitochondrial fragmentation, triggering the expression of atrophy-associated genes, proteolysis, and muscle loss. Dulac [133] et al. found that DRP1 knockdown for 4 months resulted in muscle atrophy of tibialis anterior, gastrocnemius, and finger-length extensor digitorum longus muscles, with smaller muscle fiber CSA; they also found that the DRP1 knockdown is associated with an increased FoxO3a content (93.8%), and its knockdown promoted a decrease in the expression of atrogin1 and MuRF-1. Additionally, DRP1 knockdown increased the amount of p62 as well as lipidated and non-lipidated forms of LC3B, suggesting that DRP1 knockdown prevents recycling of damaged mitochondria and affects the autophagy-lysosome system.

## Mitochondrial Quality Control and Myogenic Differentiation and Regeneration

**Figure 3.**
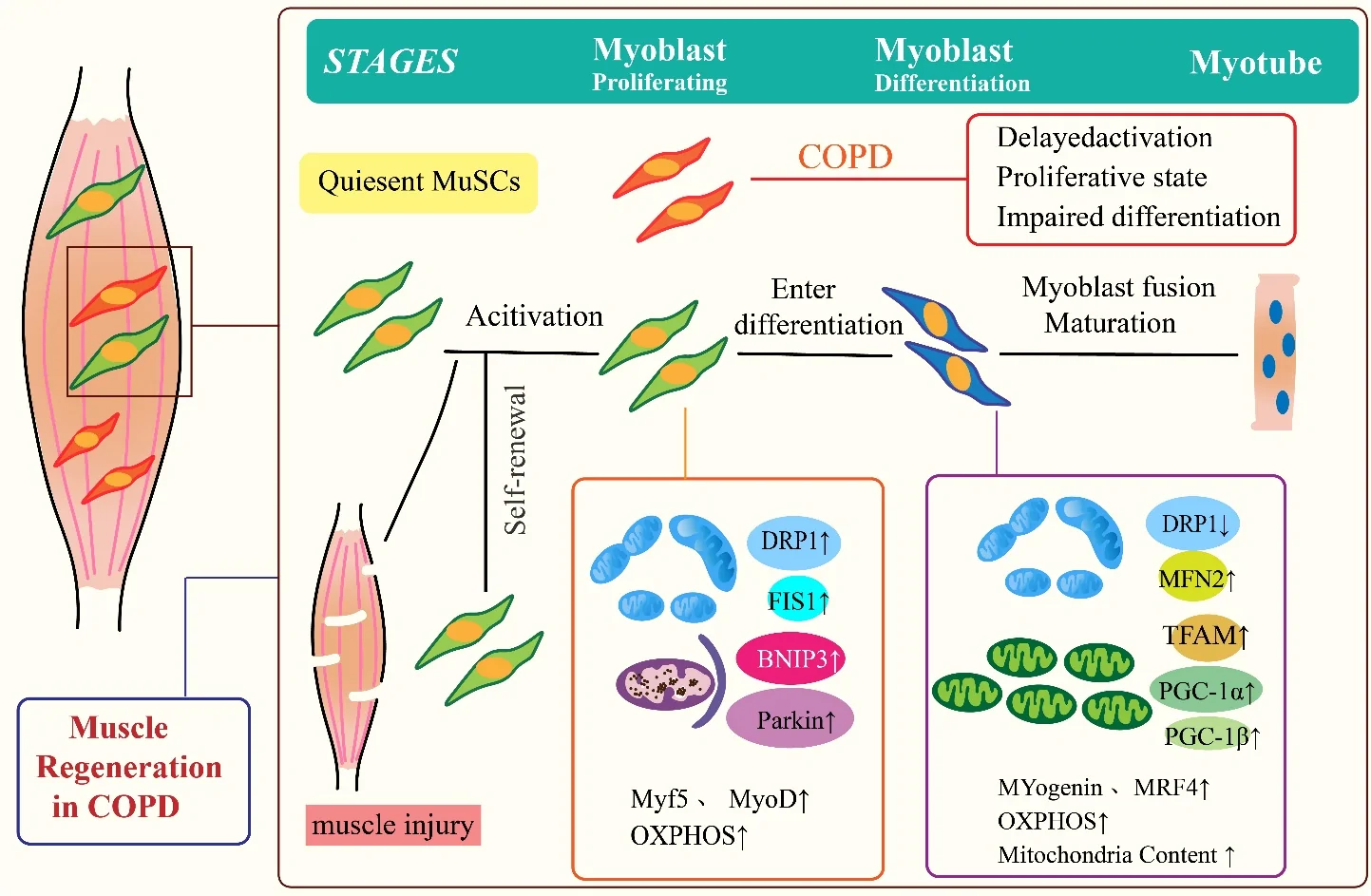
**MQC in Myogenic Differentiation and Regeneration**. Muscle injury stimulates MuSCs activation, after which MuSCs proliferate and differentiate and finally fuse to become new myotubes. In the initial phase of myogenic differentiation, mitochondrial fission and mitophagy aims to make the myoblast more oxidative, and mitochondrial fusion and mitochondrial biogenesis occur later to fuse and generate myotube. In patients with COPD, disruption and impairments in the MQC cause delayed activation of MuSCs, and they are in a proliferative state with reduced differentiation, ultimately resulting in impaired muscle regeneration.

Skeletal muscles have regenerative capacity, which is largely dependent on the MuSCs and myogenic regulatory factors (MRFs). Myogenesis is predominantly regulated by 4 MRFs: MyoD, Myf5, MYogenin, and MRF4 [134]. Myf5 and MyoD aim at specifying myoblasts for terminal differentiation, and MYogenin and MRF4 are more directly involved in the differentiation process and trigger expression of myotube-specific genes [135] (Fig. 3). MuSCs, which are quiescent skeletal muscle stem cells that are capable of self-renewal initially proliferate and then undergo myogenic differentiation after receiving a signal to activate them. After differentiation, MuSCs can fuse with injured muscle fibers to facilitate repair or differentiating and fusing with myotubes to generate new muscle fibers [136]. Patients with COPD who developed muscle atrophy have impaired muscle regeneration processes compared with healthy individuals. Patients with COPD but without muscle atrophy have a lower proportion of central nuclei (a marker of muscle regenerative events), shortened telomeres of MuSCs in muscle, which is consistent with the increased number of senescent MuSCs and depleted muscle regenerative capacity, and decreased muscle mass [137]. MyoD and Myf5 protein levels are upregulated and MRF4 and MYogenin protein levels are reduced in whole muscle extracts from patients with COPD muscular dystrophy, the activation of MuSCs from patients with COPD is delayed, and myosin heavy chain protein levels are considerably reduced during differentiation [102], suggesting that the cells are in a proliferative state with impaired differentiation during myogenesis.

Modifications of the mitochondria are necessary for the activation and differentiation of MuSCs to repair or create new myofibers. Skeletal muscle fibers have higher bioenergetic needs than myoblasts, thus myoblasts are required to renew mitochondria during myogenesis and regeneration to adapt to a more oxidative metabolic environment [138]. Decreased mitochondrial fission is one of the causes of MuSCs regenerative senescence, and mitochondrial fission allows them to induce OXPHOS to be more oxidative in response to metabolic alterations during myogenic differentiation. Mitochondrial fission is an indispensable condition for myogenic differentiation and muscle regeneration. The levels of fission factors DRP1 and Fis1 are initially substantially elevated in myoblast differentiation [139] (Fig. 3). The chemical inhibition of DRP1 reduces translocation of DRP1 to mitochondria, down-regulates fission, and suppresses the mRNA expression levels of MyoD and Myogenin in C2C12 cells. MFN2, a protein implicated in mitochondrial fusion is up-regulated during myogenic differentiation [140]. These shows disruption of mitochondrial dynamics impairs myotube differentiation and formation [141].

Mitophagy is critical in the process of skeletal muscle regeneration, and MuSCs, which induce regeneration after injury, exhibit mitophagy during proliferation and differentiation after activation [142]. Myogenic cell differentiation requires upregulation of MYogenin levels, and impaired mitophagy leads to reduced MYogenin levels in C2C12 cells, affecting myogenesis [143]. The ability to repair or regenerate in skeletal muscle of Parkin knockout mice is reduced, and MuSCs fusion is impaired, the levels of neural cell adhesion molecules (an initial marker of impaired function of MuSCs) is high, and the expression of Pax7, which maintain proliferative potential while inhibiting differentiation of MuSCs, increases, and MYogenin level is remarkably reduced. These results suggests that impaired mitophagy affects the function of MuSCs, resulting in decelerated myogenesis and incomplete differentiation [144]. MuSCs can differentiate without Parkin. At early stages of differentiation, mitophagy in myoblasts may be primarily receptor-mediated to respond to metabolic changes (Fig. 3). Increased susceptibility to apoptosis and poor differentiation were noted in BNIP3^-/-^ myoblasts [48]. It was discovered that mitochondrial fission and mitophagy occur early in the myogenic differentiation, and mitochondrial fusion and mitochondrial biogenesis are important for myofiber fusion and myotube fusion in the middle and final phases [145] (Fig. 3). During differentiation, increased expression of genes essential for mitochondrial biogenesis (e.g., PGC-1α, PGC-1β, TFAM, and NRF1, NRF2) are accompanied by increase in mitochondrial proteins and mitochondrial mass (Fig. 3). Additionally, the down-regulation of PGC-1α in C2C12 adult myoblasts leads to poor differentiation, suggesting that mitochondrial biogenesis is tightly associated with myogenic differentiation [146]. These suggest that MQC is a key factor in skeletal muscle recovery and may affect MuSCs function in the myogenic differentiation stages, leading to impaired ability to regenerate and to adjust to metabolic changes (Fig. 3).

## Mitochondrial Quality Control and Skeletal Muscle Fiber Transformation and Angiogenesis

Multiple studies illustrated a strong interconnection between mitochondrial oxidative capacity and muscle fiber-type composition [147]. The number and function of mitochondria vary according to fiber type, and mitochondrial density and number determine the oxidative capacity of muscle.

The major regulator of biogenesis, PGC-1α, regulates skeletal muscle fiber type conversion, glucose transmembrane transport and lipid utilization, and to modulate skeletal muscle oxidative capacity [148]. Skeletal muscles in humans are mainly classified into three types, exhibiting difference in fatigue resistance and oxidative potential. In type I [149], slow muscle fibers, the mitochondrial density is highest and thus leading to an enhanced oxidative capacity and resistance to fatigue. Types IIa and IIb and IIx are fast-twitch fibers, and IIx relies predominantly on glycolytic and anaerobic metabolism, fatiguing the most quickly [149]. Muscle fiber type shifts in COPD [113]. Type I fibers in the lateral femoral muscle are significantly lower (less than 27% of the total fibers) in GOLD stage 3 and 4 in patients with COPD [150]. Reduced mitochondrial biogenesis, transition from oxidative to glycolytic muscle fibers, and decreased muscular endurance occur in muscle fibers of PGC-1α knockout mice [151], which is consistent with features found in the quadriceps of patients with COPD. Resveratrol, a therapeutic drug targeting PGC-1α [152], can upregulate PGC-1α via the SIRT1-AMPK pathway, inducing an increase in mitochondrial density and a shift to oxidized fibers in myofibril types in mouse C2C12 myotubes [153]. O'Hagan [154] et al. demonstrated artificially stimulated the mitochondrial biogenesis in human primary myotubes result in increased oxygen consumption and decreased intracellular oxygen concentration, thus activate hypoxia-inducible factor (HIF), and up-regulating the expression of angiogenesis program genes. HIF-1 plays a role in the activating the factors related to angiogenesis and erythropoiesis, contributing to the promotion of vascular development, heme synthesis, and red blood cell generation. Arany [155] et al. illustrated that PGC-1α can activate vascular endothelial growth factor (VEGF) through direct binding and co-activation of ERRα at a conserved site on the VEGF promoter in a way that is HIF-1α-independent. PGC- 1α/β, ERR-induced regulator, muscle 1 (Perm1), a muscle-specific gene, plays a crucial role in the expression of genes related to muscle mitochondrial biogenesis and oxidative capacity. Notably, Perm1 is indispensable for the enhanced oxidative capacity triggered by PGC-1α in cultured myotubes. The knockdown or silencing of endogenous Perm1 [156]decreased PGC-1α-induced increase in mitochondrial DNA. Besides, Perm1 knockdown inhibited the ability of PGC-1α to augment the RNA expression of the mitochondrial DNA-encoded genes CoxII and CoxIII and nuclear genes encoding for OXPHOS components, such as CoxIV. The enhancement of Perm1 [157] by adeno-associated virus (AAV) vectors in specific muscles substantially increased mitochondrial biogenesis and oxidative capacity, and AAV-Perm1-transduced muscles showed amplified capillary density and fatigue resistance (33% and 31%, respectively). These indicated that impaired regulatory of mitochondrial biogenesis, as regulated by PGC-1α and others, is one of the major reasons for the decreased oxidative capacity of skeletal muscle in COPD.

The relationship between fiber type and mitochondrial morphology has been elucidated. The glycolytic fiber types exhibit punctate and dispersed mitochondria with little or no fusion [117]. In contrast, mitochondria in oxidative fiber types is elongated and interconnected with a high rate of fusion. Mitochondrial fusion is linked to the OXPHOS capacity of muscle fibers. Alexandre Houzelle [158] examined the proteins regulating mitochondrial fission in skeletal muscle among four distinct participant phenotypes, uncovering a positive relation between mitochondrial fusion to oxidative capacity and insulin sensitivity. Mitochondrial networks are more fragmented in low oxidative skeletal muscle (diabetes patients). Mitochondrial morphology is compatible with muscle fiber type, and the mitochondrial network is adapted to fiber-specific energy demands [159], which may be the relationship between mitochondrial dynamics and muscle oxidative capacity. Further studies are needed to decode whether targeting the mitochondrial dynamics regulatory proteins could be a strategy for improving muscle oxidative capacity.

Studies on the association between mitochondrial autophagy and skeletal muscle oxidative capacity are scarce, but from other disease models we may explore the relationship among autophagy, angiogenesis and oxidative capacity. Autophagy is crucial for potent therapeutic vascularization, particularly in cardiovascular disease such as coronary artery disease and myocardial infarction. One current study [160] explored the benefits of rapamycin on endothelial cells under high glucose conditions, the benefits including improved cell survival, inhibited oxidative stress, stimulated migratory capacity and tubulo-genesis, and autophagic modifications. These protective impacts are accompanied by the accumulation of LC3II and Beclin-1 and the degradation of p62, suggesting possibly mediated by autophagy-dependent pathway. Mizushima [161] et al. proposed a significant association between fiber type and autophagy in skeletal muscles of mice. Mofarrahi [162]et al. investigated the degree of autophagy and oxidative capacity in skeletal muscles, and found that the content of BECN1 complex, LC3B, GABARAPL1, BNIP3, and PARK2 were relatively high in with high-oxidative muscles, most of which are involved in mitophagy, suggesting in mitophagy, muscles with high mitochondrial density and oxidative capacity have a greater demand for these proteins to maintain normal MQC than muscles with low mitochondria density and oxidative capacity.

## Mitochondria-Related Treatment Strategies for COPD

### Pharmacological Approaches

The altered expression of mitochondrial dynamics proteins is partially responsible for pathological changes associated with skeletal muscle in COPD. Restoring balanced mitochondrial fusion and fission through genetic and pharmacological approaches has been demonstrated to improve tissue function in multiple models, and the protective effects observed in these models support that targeting the imbalance of mitochondrial dynamics can be a promising therapeutic option [96, 163]. The chemical inhibition of mitochondrial division by mdivi-1 prevents CS-induced airway dysfunction in COPD mice [164] and increases skeletal muscle contractile strain and oxidative capacity [165]. P110 [166], a specific peptide that regulates DRP1 activation under pathological conditions and disrupts the interaction between DRP1 and Fis1 without influencing DRP1 physiology, reduces myocardial mitochondrial fragmentation, prevents the decline in cardiac function, and reduces mortality in mice [167]. In addition to targeting mitochondrial fission, promoting mitochondrial fusion activity is not a bad strategy. Leflunomide is an activator of MFN2 [168]. BGP-15 facilitates mitochondrial fusion by activating OPA1 [169]. Considerable reduction in ROS and mtROS, and positive changes in mitochondrial network in CSE-exposed AT II cells after treatment with leflunomide and BGP15 [169] suggest that up-regulating MFN2 and OPA1 may be a novel and hopeful therapeutic choice for delaying or reversing the progression of COPD, but the relevant applications have not yet addressed skeletal muscle function.

Skeletal muscle mitochondrial biogenesis is markedly reduced in patients with COPD, making PGC-1α an attractive target for therapeutic intervention. Curcumin [170, 171]improves COPD lung and skeletal muscle function, and the mechanism may be associated with SIRT1, which up-regulates PGC-1α, promotes mitochondrial biogenesis, and regulates mitophagy to alleviate mitochondrial damage. Glycyl-l-histidyl-l-lysine-Cu2^+^ [172]was also observed to improve skeletal muscle function with increased mitochondrial density in COPD skeletal muscle through promoting mitochondrial biogenesis via the SIRT1-PGC-1α signaling pathway. In addition, some targeted PPARα/γ agonists remarkably improve lung function in COPD [173], and the beneficial effects in other disease models with impaired mitochondrial function and muscle dysfunction have been identified [174-176].

Bufei Jianpi granules [177] improve lung and muscle function, diaphragm tone and tolerance, alleviated pulmonary and systemic inflammation, and relieved mitochondrial dysfunction in rats with COPD. This effect may be related to the stimulation of mitochondrial biogenesis and inhibition of mitophagy [178]. Resveratrol [179] has been thought to activate PGC-1 to improve mitochondrial function, but supplementing resveratrol [180] in patients with COPD does not have beneficial effects on mitochondrial biogenesis and muscle function. It appears to regulate mitochondrial number and skeletal muscle phenotype by modulating mitophagy rather than mitochondrial biogenesis [181]. NAD^+ [182]^ improves mitochondrial health, muscle strength, and exercise by regulating the coordination between mitochondrial biogenesis and mitophagy. NAD^+^ [183] has multiple major precursor compounds. Clinical trials have demonstrated that 21-day nicotinamide riboside (NR) [184] supplementation has a favorable anti-inflammatory effect on the skeletal muscle in older men, and a set of genes linked to glycolysis and mitochondrial function are down-regulated but mitochondrial respiration, citrate synthase activity, and mitochondrial copy number are not altered, which may be related to increased mitochondrial quality control pathways, but the current NAD^+^-related use in COPD is still insufficient. Vitamin D supplementation [185] promotes mitochondrial biogenesis and fusion, ameliorates mitochondrial morphology and dysfunction by modulating MFN1/2, OPA1, and DRP1, and improves skeletal muscle atrophy in COPD and oxidative function.

Clinical interventions targeting MQC for the treatment of SMD in COPD remain challenging: we need to carefully monitor the impact of hypothetical interventions on muscle mitochondrial function to prevent potentially serious adverse events. MQC pathways are not isolated or “all or none”, but are interconnected with each other, having an optimal equilibrium, where both defects and excesses are detrimental [46]. Excessive mitophagy may trigger apoptosis, and even the blind up-regulation of mitochondrial biogenesis is maladaptive [186].

## Exercise Interventions

Exercise intervention has become the main nonpharmacologic intervention for skeletal muscle rehabilitation in COPD. Muscle mitochondrial disruption, low mitochondrial density, and poor oxidative capacity present in patients with COPD or animal models can be reversed by exercise to some extent [187, 188]. Current research has identified a range of exercise interventions to improve SMD in COPD, including resistance, endurance and functional training, traditional Qi gong, and combined training programs [188-190]. Wang [110]et al suggested the differences in the roles of distinct physical activities or exercise trainings to improve SMD in COPD in terms of mitochondrial biogenesis and mitochondrial redox state, and recommended that exercise programs should be selected according to disease severity and exercise capacity.

Exercise-based interventions are friendly but challenging for some COPDs, based on this, exercise simulation is an emerging possible intervention [191]. It aims to ameliorate the deficits of patients in physical performance [192]. Targeting PGC-1α is one of the pathways, and the myokines released from skeletal muscles during exercise, engaged in “crosstalk” with other organs or tissues, is also a new direction [193]. Based on mimicking exercise theory, some new exercise mimetic drugs, such as REV-ERB ligand, MOTS-C [194], PPARβ/δ agonists and ERR agonists [195] have been produced. The REV-ERB ligand regulates the gene network in charge of mitochondrial biogenesis (such as the AMPK-SIRT1-PGC-1α signaling pathway) as well as reduced the mitophagy, generally improves the oxidative capacity of skeletal muscle [196].

Numerous difficulties currently exist in the implementation of mimetic exercise measures, and the vast majority of skeletal muscle mimetics have not been tested in humans to date. In addition, patient compliance and tolerance to increased medications have not been addressed.

## Summary and Prospect

There are numerous factors contributing to impaired skeletal muscle structure and function in COPD, and among them, mitochondrial dysfunction is the core of potential pathogenetic mechanisms that affect muscle energy metabolism and performance. In this review we explore the effects and possible mechanisms of MQC in COPD with SMD, emphasizing MQC as a potential therapeutic target for COPD SMD. However, MQC are heterogeneous networks based on interaction pathways, the relevance of each of them to SMD is unclear and the dynamic regulation between them poses a considerable research challenge. Furthermore, the pathophysiology of COPD is intricate, SMD in COPD is not simple sarcopenia, and a variety of primary etiologies and pathological stages should be considered. Although alterations in muscle MQC have been found in rodent models, changes in the human patients with COPD have yet to be thoroughly explored. To date, no single model exists that can comprehensively mimic the full spectrum of phenotypes observed in human COPD [120]. More studies are needed to unravel the complexity of mitochondria controlled cellular pathways and the role of these processes in the pathogenesis in SMD of COPD.
